# Supplementary material for: A confirmation bias in perceptual decision-making due to hierarchical approximate inference
Source: PLoS Comput Biol. 2021 Nov 29;17(11):e1009517. doi: 10.1371/journal.pcbi.1009517 (PMC8659691; doi:10.1371/journal.pcbi.1009517)
Supplement: Attachment — Submitted filename: Reply2Reviewers.pdf [file pcbi.1009517.s002.pdf]

Responses to reviewer comments

Richard D. Lange, Ankani Chattoraj, Jeffrey M. Beck, Jacob L. Yates, Ralf M. Haefner

September 19, 2021

Summary

We thank the reviewers for their constructive criticisms. The original reviews are colored below in blue, and our replies are in black.

The most important changes with this revision are:

- Better justification of why the evidence accumulation dynamic that we describe constitutes a confirmation bias.
- A more detailed and comprehensive discussion of our work with respect to related, prior studies.
- A more detailed explanation for how the strength of the confirmation bias dynamic that we describe relates to the amount of category information.

Reviewer 1

This paper tackles a puzzling effect in the literature: why do temporal integration biases (recency vs. primacy) differ across studies of perceptual decision-making? The authors propose an elegant, unified solution where the main driver of such biases is the relative amount of category vs. stimulus noise in each sample of evidence. This follows from a model of hierarchical approximate inference, where the effect of the category prior on sensory likelihood drives primacy effects ('confirmation bias'). Recency effects can be produced when an additional leak term is added to the model. The authors then show that this model can better account for the data than established leaky temporal integration.

This is beautiful work that ties together a great many studies from the literature, and will surely influence how the field thinks about the shape of psychophysical kernels. The paper is well written, the data crystal clear, and the results appealingly presented (I appreciate the indication of which datapoints are authors in Figure 5). I unfortunately lack the mathematical expertise to validate the derivations of the inference model; however, I fully trust the author's expertise in this domain. I was impressed when I first saw this presented at a conference a few years ago, and strongly support publication of this work.

We thank the reviewer for their positive evaluation of our work.

I only have a few small suggestions which may further improve this excellent paper.

- In the intro or discussion, it would be good to add a short discussion of Keung et al. (https://doi.org/10.1038/s41467-020-15630-0) who propose that divisive normalization can account for different kernel shapes.

We thank the reviewer for drawing our attention to this interesting and relevant paper that we had missed. We now cite this paper in both Introduction and Discussion as suggested. In brief, this paper presents a version of a "race-model" which – like other race models, the drift-diffusion-model, and attractor-models of decision-making – describe the dynamics of decision-making *within* the decision-making circuits. As we describe in the Discussion, these models are all complementary to our work, which models the effect of the influence of the decision-making area on sensory representations. The actual weighting applied by the subject and observed by the experimenter in any real-life context will be a combination of multiple elements, with the dynamic described by us being one of several.

Specifically, it would be interesting to discuss non-linear 'bump' kernels described in this paper, which may be present in some observers in Figure 5a.

Thank you for the suggestion – that is an interesting observation. However, we are very reluctant to overinterpret the nonmonotonic shapes seen in individual observers in our dataset since we think they are mostly sampling noise despite thousands of trials going into each curve for two reasons: (1) monotonic linear and exponential kernels actually had the highest cross-validated likelihood for all subjects, given the data, and (2) the deviations from monotonicity are a lot smaller in the HSLC than the LSHC condition – mirroring the smaller uncertainty (errorbars) around the individual kernels in the HSLC compared to the LSHC condition.

Could your model account for such unusual shapes?

The feedback dynamic between decision & sensory areas that we describe cannot, by itself, explain non-monotonic shapes. However, as we emphasize in the Discussion, the dynamic described by us is complementary to other aspects of the perceptual decision-making system, such as dynamics *within* either sensory or decision-making circuits. In our own experiments (part of a separate paper in preparation), we have observed bump-shaped kernels in situations when participants needed extra time to relocate their endogenous attention to parts of the visual space that contained the signal, leading to a brief ramp before a classic primacy shape of the weighting profile.

l. 145: "... which in turn is determined by the category information in the task". This does not naturally follow - could you add an intuition for how the influence of the prior depends on the stimulus vs. category information in a specific task? Only when reading the legend of Figure 2 repeatedly did the effect of the category information on prior effect become clearer, it may be helpful to briefly state the intuition upfront.

Thank you for pointing this out. We agree and have now expanded that sentence into a whole paragraph explaining this important point.

The revised version now reads: "Importantly, the strength of the prior only depends on the amount of category information, and *not* the amount of sensory information (unlike task performance which depends on both). For instance, in a task with high category information such as the classic dot motion task (Newsome & Pare 1988), high certainty in the trial category based on stimulus frames seen so far within a trial, directly translates into high certainty in the observer's expectation about the subsequent stimuli within the trial (Figure 2b). In a low category informa-

tion task such as the Poisson clicks task (Brunton et al. 2013), on the other hand, knowing the trial category is only weakly predictive whether any one click is on the left or on the right (Figure 2a)."

I find the abbreviations LSHC and HSLC rather confusing, taking a lot of working memory to remember their placement in the space in figure 1c. Please consider replacing them with something easier to parse (perhaps 'sensory noise dominated' vs. 'category noise dominated').

We feel the reviewer's pain, and we have indeed long agonized over the best way to abbreviate those two conditions. The problem is that some readers prefer to think in terms of information, others in terms of noise (inverse relation, of course); and some find the labeling by what is high more intuitive, and others by what is low. We have tried to deal with this by consistently using the colors red and blue throughout all figures and some readers in fact preferred to think of the 'red' and the 'blue' condition, respectively. In order to reduce the cognitive load further we have now colored all "LSHC" red throughout the text, and all "HSLC" blue.

l. 257 and 267: 6c → 6b (?), and l. 277: 6c → 6b

We thank the reviewer for pointing out these typos which we have corrected in the revised version.

Reviewer 2

The heterogeneity in temporal weighting profiles in laboratory decision-making experiments has been well-documented. Yet little is known about the factors governing this heterogeneity. In this article titled "A confirmation bias in perceptual decision-making due to hierarchical approximate inference", Lange and colleagues addressed this question suggesting that statistics of the stimuli presented during the task is one such factor. They provided support to this hypothesis with a qualitative clustering of previous studies, and a combination of novel psychophysics task manipulation with human observers and Bayesian modelling.

The article offers a fresh perspective towards the variety of temporal weighting profiles observed in decision-making studies. I believe the perceptual decision-making community finds this insight very relevant, and interesting. I congratulate the authors on a study well-done. I have a few comments most of which can be addressed by revisions to the manuscript without the need for any additional analyses.

We thank the reviewer for their positive evaluation of our work.

Major concerns: 1. The authors use the term "confirmation bias" rather gratuitously throughout the article. It has a widely accepted definition in psychology as the tendency to selectively seek or interpret information to support existing beliefs or hypotheses (Nickerson, 1998). But this is not what the authors imply when they say "perceptual confirmation bias", but rather a mechanism by which an under-corrected prior lead to primacy. As far as I know, a primacy effect is not selective (where supporting evidence is overweighed or conflicting evidence in underweighted), but a general tendency to ignore evidence once a decision (or a bound, in terms of integration to bound framework) is reached. I would suggest the authors to drop it completely: the article will not meaningfully change as a result of this. If the authors feel strongly about keeping the term, then please clarify how their definition of the term is similar to the generally accepted one.

We thank the reviewer for pointing out the need for justifying our use of 'confirmation bias' – which is indeed a key element of our work. We have now done so in both the Abstract, and the Results section, and elaborated the connection between our work and that of related studies on the confirmation bias as suggested by the reviewer.

In brief, incorporating temporal priors biases the interpretation of new evidence in accordance with existing beliefs, since the prior reflects evidence accumulated part-way through a trial. As we say in the paper, simply including a prior does not lead to biases when inference is exact and can later account for it. "Double-counting" or "under-correcting" the prior causes a bias in the decision through a self-confirming positive feedback loop. Together, these constitute a "selective interpretation of information to support existing beliefs," and in fact suggest a bounded-optimality rationale for the origin of such a selective interpretation process. Throughout, we use the term "primacy effect" to refer to patterns in the data, and "confirmation bias" to refer to the proposed positive feedback loop mechanism, and we wholeheartedly agree that many different mechanisms could in principle underlie primacy effects, and not all primacy effects are due to confirmation biases. This is why our model-fitting and ablation analysis is important: it shows that the positive feedback loop mechanism (which is consistent with our use of "confirmation bias") describes the data better than bounded integration (which results in primacy effects but which would not satisfy the above definition of a confirmation bias).

We now write in the revised Abstract, "The key dynamic that leads to a primacy bias in our model is an overweighting of new sensory information that agrees with the observer's existing belief – a type of 'confirmation bias'."

The most relevant part of the Results section now reads: "*Under*-correcting for this prior would lead to earlier frames entering into the update multiple times, giving them a larger weight in the final decision. Over multiple frames, the effect is a positive feedback loop between estimates of sensory features x_f and the belief in C. This mechanism constitutes a "perceptual confirmation bias," since belief updates are biased towards confirmatory evidence (Nickerson 1998, Michel & Peters 2020), and leads to primacy effects."

If the authors choose to do this, please spend some space in the discussion relating this work to recent studies investigating confirmation bias in perceptual decisions (Talluri, Urai et al. 2018 Current Biology, and Rollwage et al. 2020 Nat Comm. come to mind).

We agree that these studies are related in spirit which is why the original manuscript already contained a discussion of Talluri, Urai et al. 2018 (lines 360-366 in the original manuscript). We have now added Urai et al. 2019, Rollwage et al. 2020, and Talluri et al. 2021 to that discussion in the revised version. What all these studies have in common with each other, and with Stocker & Simoncelli 2007, and how they differ from ours, is that the observed bias is linked to a choice, i.e. an action taken by the observer. Furthermore, none of these studies provides an explanation derived from normative principles, or one which can predict when this bias should be strong and when it should be weak based on the statistics of the stimulus itself.

We thank the reviewer for the reference to Rollwage et al. which we had overlooked. We have now also added a Discussion paragraph that specifically addressed the relationship between subjective confidence and confirmation bias pointed out by Rollwage et al. and that also exists in our work.

2. Related to the point above, the title of the article is misleading – I started reading the paper expecting insights into confirmation bias, and found myself wondering how the article adds to our understanding of this bias. I would suggest the authors to use a title more appropriate to what the article is about – which is temporal weighting biases.

We hope our arguments above have convinced the reviewer that our work indeed revealed a kind of confirmation bias. If not, we welcome a more specific critique as to why the reviewer thinks that our model does not describe a kind of confirmation bias through selective interpretation.

We are also not aware of previous work that links the "selective interpretation" type of confirmation bias to approximate hierarchical inference. We agree that further work is needed to translate the hierarchical inference dynamics from our models into other domain(s) where confirmation biases have primarily been studied, but we hypothesize that the basic principles of our models – such as over- and under-correcting for a prior as well as how this interacts with the decomposition of information into two types – will translate effectively (see final paragraph of our Discussion).

3. The article does not mention (or only does so sparingly) some of the recent studies on flexible temporal weighting strategies in perceptual decisions within individuals. At least three other studies come to mind: Levi et al. 2018 eNeuro (doi: 10.1523/ENEURO.0169-18.2018) Bronfman et al. 2016 PLoS Comp Biol. (doi: 10.1371/journal.pcbi.1004667) Talluri et al. 2021 J. Neurophys. (doi: 10.1152/jn.00462.2020) Given the relevance between these studies and the current one, I urge the authors to dedicate a paragraph in the introduction giving an overview of the state of the art in temporal weighting biases in decision-making studies, and how the current study addresses the gaps unfilled by these studies. Furthermore, the similarities and differences between the current article and these studies warrants a paragraph in the discussion.

We thank the reviewer for pointing out these studies. Even though one of us is a co-author on

Levi et al. 2018, we do not think it is paricularly relevant to our work since it explicitly addresses changes in the temporal weighting in response to stimuli whose signal is varied over time. However, we now cite the other two studies suggested by the reviewer in Introduction and Discussion. In brief, Bronfman et al. 2016 proposes as a variant of accumulation dynamics *within* decision circuits (as e.g. Usher & McClelland 2001 and the attractor-based models) and is covered by the corresponding paragraphs. Talluri et al. 2021 advances the work on weighting-changes due to choices (following Stocker & Simoncelli 2007, Luu & Stocker 2018, and prior works by the Donner lab) and is now cited in the corresponding paragraph.

For example, does the approximate hierarchical inference also account for the findings in these previous studies? Can the dynamic LCA model proposed to explain the empirical findings in Bronfman et al. 2016 also account for the empirical findings in the current article?

The key insight of our modeling – that hierarchical approximate inference induces a positive feedback loop between decision-making and sensory areas that in turn leads to a confirmation bias – can only explain a primacy effect whose strength depends on the balance of category and sensory information in the stimulus, and not, e.g. a primacy in the beginning and a recency at the end of a very long trial (Bronfman et al). However, as emphasized in the Discussion, the dynamic described by us acts independent/in addition to any cognitive or decision-specific mechanisms. As such it can be combined with any other model that focuses e.g. on learning (like Levi et al.), or proposed ad-hoc mechanisms how a change from primacy to recency may be caused on a neural level (Bronfman et al.), or what the effect of committing to a choice is on the decision-dynamics (Stocker, Talluri, Urai & colleagues). Of these, our work is most closely related to the latter studies, and we have now expanded our Discussion that elaborates on a possible extension of our work to make closer contact with them: "these previous findings on the effect of commitment to a choice on weighing subsequent evidence can easily be accommodated in our framework by plausibly proposing that the act of committing to a choice increases one's subjective certainty about that choice being correct. In our model, such an increase in certainty would directly translate into an increase in feedback from decision to sensory areas, and hence increased confirmation bias."

With regards to Bronfman et al. who explained a combination of early and late weighting in very long trials with a specific neural adaptation process: our work demonstrates that it is not LCA that dominates the primacy effect (otherwise its strength would not depend on the balance of sensory and category information), and hence suggests that the transition from primacy to recency might not be best-explained by LCA+adaptation. But since our data cannot speak to that question, we prefer to not do so either in our paper.

4. Lines 177-179: "While the exact magnitude of the leak is a free parameter in our model, to be constrained by data, the change in bias with changes in category information is a strong prediction…". If the leak parameter was indeed fit to the empirical data from the experiment, then please plot this for all subjects as a subfigure in figure 5. This was shown for the extended ITB model in figure 6, but not for the approximate hierarchical inference model.

If we understand the reviewer correctly, then they might be misunderstanding what we have done. We did not fit our hierarchical approximate inference model directly to the data. Instead, we first show that it is functionally equivalent to an extended drift-diffusion model. We then add a bound to that model resulting in what we called the 'extended integration-to-bound' model. By virtue of containing a parameter determining integration dynamics, and a parameter representing a bound, this model can explain a primacy effect in the data by either or both mechanisms. Fitting this model to the psychophysical kernels only would not be able to distinguish between

both mechanisms since they can both explain a decreasing kernel. However, fitting this model to individual subject choices allows us to determine which of the two proposed mechanisms *actually* accounts for the observed primacy effect. By treating both mechanisms equally, giving both the same number of parameters, the same link between sensory data and decision-variable, etc. we aimed to make this comparison as fair and rigorous as possible.

We also note that both models – our approximate inference model and the extended ITB model – have a "leak" term. However, while the leak in the approximate inference model is strictly positive, modeling forgetting, the "leak" parameter in the extended ITB model combines the effect of a confirmation bias and the effect of forgetting since they are not distinguishable based our data. If the confirmation bias dominates, it will be negative (overall acceleration), if the forgetting dominates, it will be positive (overall deceleration, i.e. a classic leak). Figure 5 shows the inferred posterior distributions over the leak and bound – in the second model, which is the only one we fit to the data – for each subject.

Minor concerns:

1. This is mostly about semantics, so the authors can choose to ignore it: Primacy/recency biases have been demonstrated in non-perceptual paradigms as well (for eg., Peterson & DuCharme 1967 J Exp. Psych.; Tsetsos et al. 2012 PNAS; Bronfman et al. 2015 Proc R Soc B; Tsetsos et al. 2016 PNAS; Talluri et al. 2021 J Neurophys., to name a few). It helps with the generalizability of the model to these paradigms if the authors replace "sensory information" with another term (like "feature information", reflecting that features of the stimulus used for the decision-making process) that is not very grounded in the perceptual paradigm.

We thank the reviewer for this excellent point. We absolutely share the reviewer's intuition that the dynamic uncovered by our work is present in non-sensory domains – possibly whenever approximate inference is performed in hierarchical systems. For instance, when forming an opinion about the benefit of certain policies in the context of a pandemic by following the news, the observer sequentially updates their beliefs on the basis of individual reports, many of which are associated with some uncertainty about their trustworthiness/representativeness/etc. ("sensory information") and with different degrees of relevance for the belief about whether a specific policy is beneficial ("category information"). More general terms would certainly be needed in a general context. However, since our work is specifically concerned with perceptual decision-making, tested using a low-level perceptual decision-making experiment, and written for a sensory processing audience with the hope to inspire future studies testing its neurophysiological predictions (see Discussion), we have not been able to come up with a more intuitive term than "sensory information". "Feature information" for instance might leave it uncertain whether it refers to the information of the feature for the category, or the information in the sensory inputs for the feature. We feel that "sensory/category" minimizes the risk of confusing which of the two corresponds to the information at the lower level, and which corresponds to the information at the higher level of processing.

2. Lines 134-135: "instead, the decision-making area now needs to account for or "divide out" the influence of the top-down prior on the sensory representation (Figure 2b-c)." This sentence sounds like a decision-making area in the brain actively discounts the prior to prevent double counting. Please justify this by citing any relevant past work, or clarify if this is one of the predictions of the current article.

We thank the reviewer for pointing out this misunderstanding. We do not claim that it does (which would indeed require a justification), but merely that it would (hypothetically) need to in order to not miscalculate. We have now made this clearer by writing "the decision-making

area would need to account for or "divide out" the influence of the top-down prior on the sensory representation to avoid a double-counting of the prior".

3. Line 358: Please cite Ossmy et al 2013 Current Biology (doi: 10.1016/j.cub.2013.04.039) which showed that humans also adapt their timescale of evidence integration to stimulus statistics.

We thank the reviewer for pointing this reference which we had forgotten to include. Now added in the revised version.

4. Please include missing citations for Deneve et al. 2012 at line 42, and Prat-Ortega et al. 2021 at line 43.

Done & done.

5. Please update references 57 & 63 to reflect their current publication status. These studies are now published.

Many thanks! Both are now updated.
